# RNomic identification and evaluation of npcTB_6715, a non‐protein‐coding RNA gene as a potential biomarker for the detection of *Mycobacterium tuberculosis*

**DOI:** 10.1111/jcmm.13148

**Published:** 2017-07-29

**Authors:** Priyatharisni Kanniappan, Siti Aminah Ahmed, Ganeswrie Rajasekaram, Citartan Marimuthu, Ewe Seng Ch'ng, Li Pin Lee, Carsten A. Raabe, Timofey S. Rozhdestvensky, Thean Hock Tang

**Affiliations:** ^1^ Advanced Medical & Dental Institute (AMDI) Universiti Sains Malaysia Kepala Batas Penang Malaysia; ^2^ Department of Pathology Johor Bahru General Hospital Johor Malaysia; ^3^ Institute of Experimental Pathology (ZMBE) University of Muenster Münster Germany; ^4^ Institute of Evolutionary and Medical Genomics Brandenburg Medical School (MHB) Neuruppin Germany; ^5^ Institute of Medical Biochemistry Centre for Molecular Biology of Inflammation (ZMBE) University of Muenster Münster Germany; ^6^ Department of Medicine (TRAM) University Hospital of Muenster Münster Germany

**Keywords:** small RNA (sRNA), *Mycobacterium tuberculosis*, multiplex PCR, diagnosis, RNomic

## Abstract

Technological advances in RNA biology greatly improved transcriptome profiling during the last two decades. Besides the discovery of many small RNAs (sRNA) that are involved in the physiological and pathophysiological regulation of various cellular circuits, it becomes evident that the corresponding RNA genes might also serve as potential biomarkers to monitor the progression of disease and treatment. sRNA gene candidate npcTB_6715 was previously identified *via* experimental RNomic (unpublished data), and we report its application as potential biomarker for the detection of *Mycobacterium tuberculosis* (MTB) in patient samples. For proof of principle, we developed a multiplex PCR assay and report its validation with 500 clinical cultures, positive for *Mycobacteria*. The analysis revealed 98.9% sensitivity, 96.1% specificity, positive and negative predictive values of 98.6% and 96.8%, respectively. These results underscore the diagnostic value of the sRNA gene as diagnostic marker for the specific detection of MTB in clinical samples. Its successful application and the general ease of PCR‐based detection compared to standard bacterial culture techniques might be the first step towards ‘point‐of‐care’ diagnostics of *Mycobacteria*. To the best of our knowledge, this is the first time for the design of diagnostic applications based on sRNA genes, in *Mycobacteria*.

## Introduction

npcRNAs (non‐protein‐coding RNAs) do not encode significant open reading frames and are not translated into proteins 1. However, these RNAs serve important regulatory roles, often in complex with proteins 2. npcRNAs are further classified according to RNA processing patterns 3 or size criteria 4.

sRNAs in bacteria represent small npcRNAs with sizes typically ranging between 10 and 500 nucleotides (nts) 2, 5. sRNAs often encode conserved RNA secondary structure or antisense elements to allow specific interactions with nucleic acid or protein targets 6, respectively. Notably, the conservation of either element does not necessarily entail appreciable primary sequence preservation. In agreement, deep sequencing uncovered copious instances of species‐specific non‐protein‐coding transcripts 5.

*Mycobacterium tuberculosis* is the causative agent of tuberculosis (TB). It was responsible for 9.6 million infections in 2014 7 and an estimated number of 1.5 million deaths worldwide 4. The analysis of global surveillance data revealed that tuberculosis infections are most widespread in Asia (58%) 7. Effective control of tuberculosis outbreaks and appropriate treatment of diseased individuals demands the timely identification of infected patients. The detection of MTB in patient samples *via* PCR is less time consuming, cheaper and provides most accurate results 8, 9. In addition, this approach requires less sophisticated laboratory equipment than the original, more demanding cell culture‐based methods.

The IS*6110* is an insertion element specific for the mycobacterial complex and is represented by up to 25 copies per genome 10. So far, the IS*6110* insertion sequence has been extensively exploited as target for multiplex PCR‐based analyses of MTB 11, 12, 13. But, of late there are reports of extreme instances where genomes of some MTB strains only contain a single copy of IS*6110* 14, while others even lost the insertion sequence entirely 15, 16. The recent emergence of IS*6110*‐negative strains particularly in Southeast Asia 17, 18, warrant for alternative targets that can complement the use of IS*6110* in a multiplex‐based assay.

Despite of the growing evidence that sRNAs can serve as biological markers for many human diseases 19, 20, 21, the application of sRNA genes as targets for diagnosis of infectious agents has so far not been scrutinized in greater detail 22. With small size cDNA libraries and intensive blast analysis, we identified sRNA gene candidates that were specific to MTB complex. Depending on sequence composition, length and G/C content, these candidates potentially represent suitable targets for the design of multiplex PCR assays in combination with IS*6110*.

To this end, we describe the experimental validation of the npcTB_6715 sRNA gene as complementing target to IS*6110* in a multiplex PCR assay for the detection of MTB. To reduce false negatives, amplification controls were designed and routinely included in the mPCR (multiplex) assay. The PCR test was validated with 500 culture positive clinical samples, and to rank its diagnostic potential, we compared the results with a standard commercial kit for TB diagnosis (Genotype Mycobacterium CM Hains line probe assay).

## Materials and methods

### Bacterial isolates and genomic DNA extraction

All bacterial strains in this study were part of the culture stock collection of the Department of Microbiology & Parasitology, School of Medical Sciences and Infectomic Cluster, Advanced Medical and Dental Institute, Universiti Sains Malaysia. DNA extraction for MTB and non‐tuberculosis mycobacteria (NTM), respectively, was carried out as described 23. In brief, the DNA extraction is based on chemical and enzymatic lysis of the bacterial cells followed by a chloroform‐isoamyl alcohol extraction. Basically, MTB cells were collected into 500 μl Tris‐EDTA buffer, pH 8.0. The cell suspension was then heated for 20 min. at 80°C. Lysozyme was added (final concentration at 1 mg/ml), followed by incubation at 37°C for 2 hrs. After the incubation, 10% sodium dodecyl sulfate (final concentration 1.1%) and proteinase K (final concentration at 0.2 mg/ml; Promega Inc. Madison, Wisconsin, USA) were added. Tubes were gently vortexed gently and incubated at 65°C for additional 20 min. Next, a mixture of *N*‐acetyl‐*N,N,N*‐trimethyl ammonium bromide (CTAB; final concentration of 40 mM) and NaCl (final concentration of 0.1 M) was added, followed immediately by the addition of NaCl alone (final concentration 0.6 M). The tubes were again vortexed before further incubation at 65°C for 10 min. Equal volume (800 μl) of chloroform‐isoamyl alcohol (24:1) was added to each tube, vortexed and then centrifuged at 12,000 × *g* for 5 min. The genomic DNA in the aqueous phase was precipitated with equal volume of isopropanol and washed with 80% ice‐cold ethanol. The DNA pellet was air‐dried briefly and resuspended in 20 μl double‐distilled water.

For cDNA library construction, MTB H37Rv was grown in Middlebrook 7H9 broth (Difco, Beckton Dickinson and Company, Sparks, MD 21152, USA) supplemented with 10% Middlebrook ADC enrichment (Becton, Dickinson and Company, Sparks, MD 21152 USA). Bacterial cultures were incubated at 37°C in a 250 ml screw cap Erlenmeyer flask and shaken manually twice per day. Cells were harvested during the lag, mid‐log, stationary and late stationary phases. The cell pellets were stored at −80°C or directly processed for total RNA extraction.

For all other bacteria (Table S2, excluding MTB and NTM), we followed an in‐house designed DNA extraction protocol. In brief, 1.5 ml of bacterial culture was centrifuged at 5000 × *g*, the supernatant was discarded and tubes were vortexed to disperse bacterial pellets, the cells were suspended in 200 μl Solution I (20% sucrose, 50 mM Tris‐HCl [pH 8.0], 1% SDS, 0.2 M NaOH, 25 mM EDTA [pH 8.0], 0.1 M NaCl).

The suspension was subsequently neutralized with 200 μl Solution II (3 M NaOAc, pH 5.2). After centrifugation at 13,000 × *g*, the genomic DNA was precipitated with 100% ice‐cold ethanol, pelleted at 13,000 × *g*, washed with 70% ice‐cold ethanol, air‐dried and re‐suspended in 50 μl autoclaved ddH_2_O. The concentration of the genomic DNA was measured *via* the NanoDrop™ 2000 system and stored at −20°C until used for PCR.

### Total RNA isolation, cDNA library construction and sequencing

The RNA isolation method is based on single‐step guanidine isothiocyanate/phenol/chloroform extraction 24. Cell pellets (100 mg) were resuspended in 1 ml of TRIzol reagent (Gibco BRL; Life Technologies, Applied Biosystems Inc., Foster City, California, USA). The suspension was then transferred to pre‐chilled 2 ml screw cap tubes containing 0.1 mm diameter zirconia/silica beads (Biospec Products Bartlesville, OK 74005, USA). Cells were homogenized with the FastPrep^®^‐24 (MP Biomedicals Irvine, California, USA) by subjecting to 3 × 1 min. pulses at 4.5 m/s with 1‐minute rests on ice during each interval. The supernatant was transferred into 1.5‐ml microcentrifuge tubes, and total RNA isolation was conducted according to the manufacturer's protocol (Gibco BRL, Life Technologies). To 1 ml of lysate was added: 0.1 ml of 2 M sodium acetate, pH 4.0 and 1 ml water‐saturated phenol. The mixture was then mixed thoroughly by inversion and 0.2 ml chloroform‐isoamyl alcohol (49:1) were added. The resulting biphasic system was vigorously shaken by hand for 10 sec. The sample was cooled on ice for 15 min. and centrifuged 12,000 × *g* for 20 min. at 4°C. The upper aqueous phase was then carefully transferred to a clean tube, and 1 ml isopropanol was added for RNA precipitation for at least 1 hr at −20°C. The resulting RNA pellet was collected by centrifugation for 10 min. at 10,000 × *g* at 4°C and washed with 1 ml of 75% ethanol. Finally, the RNA pellet was air‐dried for 5–10 min. at room temperature and solubilized in 50 μl autoclaved ddH_2_O. Total RNA isolated was then treated with DNase (Ambion; Applied Biosystems, Foster City, California USA).

For analysis of sRNA transcription in MTB, we generated small size cDNA libraries, which are enriched for candidate sRNAs. Combined total RNA (200 μg) representing the four different growth stages, lag (5 days), mid‐log (14 days), early stationary (28 days) and late stationary (50 days), were size fractionated (20–350 nts) on 8% denaturing polyacrylamide gels (7 M urea, 1× TBE buffer). Size fractionation of total RNA starting material *via* 8% polyacrylamide gel‐electrophoresis enabled the selection of RNAs with size ranges of 20–350 nts. Passive elution was conducted in 0.3 M sodium acetate, NaOAc (pH 5.3) overnight at 4°C. After passive elution, the resulting starting material was 3′ C‐tailed and linked to DNA adapters at 5′ termini according to Raabe *et al*., 25. The procedure enabled subsequent steps of reverse transcription and single stranded PCR amplification. The PCR product was then double digested with *Sal*I (Roche Applied Science, Mannheim, Germany) and *Not*I (Roche Applied Science, Mannheim, Germany) restriction endonucleases and ligated (T4 DNA ligase, Roche Applied Science, Mannheim, Germany) into the pSPORT1 cloning vector (Invitrogen Life Technologies, Applied Biosystems Inc., Foster City, California, USA). Finally, 5400 colonies were selected at random and sequenced 26. With the help of Seqman, all cDNAs were assembled into 3030 contigs, which ultimately represent all sRNA candidates of our survey. Contigs with sizes below 15 nts were excluded from downstream analysis.

### Bio‐computational screens for MTB complex specific sRNA genes and primer design

5′‐adapter and C‐tail sequences were trimmed from the cDNAs using custom UNIX scripts. cDNAs that were shorter than 15 nts were excluded from further analysis. The resulting cDNAs were assembled into contigs using SeqMan Pro 8.1.5 (LaserGene, DNASTAR, Madison, WI, USA) 27 with parameters as described by Raabe *et al*., 25. The assembled contigs were manually analysed and mapped against the MTB H37Rv genome (Refseq: NC_000962) using Basic Local Alignment Search Tool (BlastN) 28. The Blast search was conducted with E‐values of 10^−5^ and a minimal word size of 11 for the identification of sRNA genes specific for MTB complex. The NCBI Primer design software (www.ncbi.nlm.nih.gov/tools/primer-blast/index.cgi) was used to design suitable PCR primers for amplification of sRNA gene, IS*6110* and the control plasmid pL250 (Table S1).

### mPCR assay

The mPCR assay was conducted with the MyCycler™ (Bio‐Rad Laboratories, Hercules, California, USA) in a total reaction volumes of 20 μl, each PCR was supplemented with 0.25 μM of forward and reverse primer for IS*6110*, npcRNA_6715 and plasmid DNA pL250, 200 μM dNTPs (Fermentas, ThermoFisher Scientific, Waltham, Massachusettes, USA), 1.5 mM MgCl_2_ (Fermentas ThermoFisher Scientific, Waltham, Massachusettes, USA) and 2 U *Taq* DNA polymerase (Biotools, Madrid Spain) in 1× PCR buffer (10 mM Tris‐HCl, [pH 8.3], 50 mM KCl). One ng genomic DNA served as template for PCR amplification and 10 ng plasmid DNA pL250 were employed as amplification internal control (AIC) to rule out false negatives caused by potential PCR inhibitors.

The PCR parameters: initial denaturation at 95°C for 1 min., 30 amplification cycles (i.e. 30 sec. denaturation at 95°C, 30 sec. annealing at 66°C, 30 sec. extension at 72°C), with final elongation step of 2 min. at 72°C. The PCR conditions were optimized with varying concentrations of primers, MgCl_2_, dNTPs and AIC. In addition, annealing temperatures were optimized by gradient PCR. The specificity of the mPCR assay was determined with 10 NTM species, 7 respiratory tract‐associated pathogens and 7 normal flora bacteria (i.e. bacteria that would represent non‐diseased physiological conditions) (Table S2). The analytical sensitivity of the mPCR assay was determined based on the lowest amount of DNA template per reaction that yields a PCR band of the expected size in agarose gel electrophoresis.

### Gel electrophoresis

All PCR products were analysed by gel electrophoresis. Twenty microlitres of each amplified sample was loaded on a 3.5% agarose gel and electrophoresed in 1× TAE buffer at 60 V for 1 hr. Gels were stained with ethidium bromide and visualized using a gel‐imaging system (Bio‐Rad, USA). PCR product from the samples was randomly chosen and sequenced 26.

### Line probe assay (LPA)

The Line probe assay (GenoType Mycobacterium CM, Hain Lifesciences, Nehren, Germany) is a molecular test for the identification and differentiation of the MTB complex and common NTM 29. The assay amplifies the 23S rRNA gene, followed by reverse hybridization to specific oligonucleotide probes for different mycobacterial species immobilized on membrane strip. Hybridization events were detected *via* colorimetric changes mediated by the enzymatic reaction of the streptavidin‐conjugated alkaline phosphatase against the substrate.

The identification of individual mycobacterial species relies on the detection of specific banding patterns. LPA analysis was performed according to the manufacturer's recommendations. In brief, PCR amplifications were conducted in 50 μl reaction volume, comprising of 35 μl of primer‐nucleotide mix, 5 μl of 10× amplification buffer containing 2.5 mM MgCl_2_ and 1.25 U of hot start *Taq* polymerase. Five μl genomic DNA (15 ng/μl) and distilled water were added to adjust the final reaction volumes. The amplification products were denatured in a denaturing reagent (provided with the kit) at room temperature, followed by hybridization at 45°C for 30 min. in a shaking water bath (Memmert, Germany). After two washing steps, streptavidin conjugated‐alkaline phosphatase and the substrate were added for the colorimetric reaction. To control for cross‐contamination, sterile double‐distilled water in lieu of DNA template was added to the negative control included for each run. (For details regarding buffer compositions: http://www.hain-lifescience.de/en/products/microbiology/mycobacteria/genotype-mycobacterium-cmas.html).

## Results and discussion

### Analysis of cDNA library

**Figure 1:**
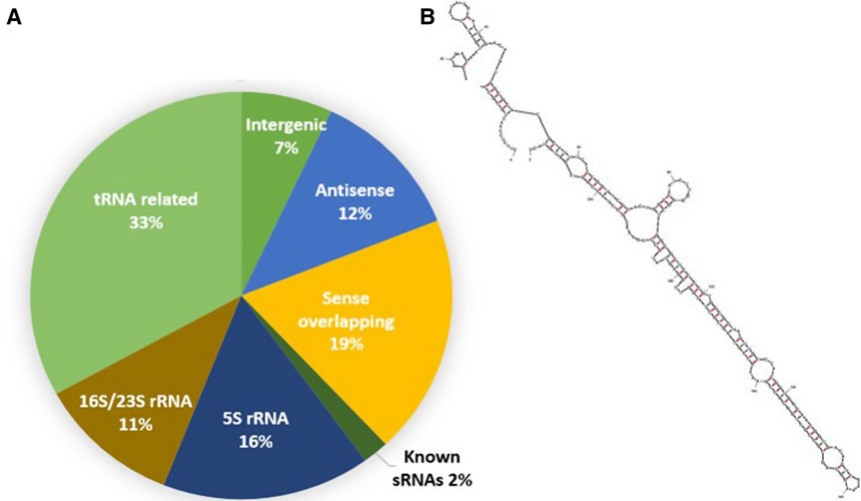
(**A**) Distribution of RNA species from 4093 clones from cDNA sequences. cDNA sequences were grouped according to their annotation and overlap to known features within the genome. Indicated percentage denote the relative number of cDNAs within our library. (**B**) Predicted secondary structure of npcTB_6715.

About 19% of all sRNAs within our surveys were derived from protein‐coding mRNAs. Here, the majority of candidate contigs partially overlapped 5′ and 3′ termini of annotated CDS regions. Notably, we also uncovered small antisense transcripts, which reside on the reverse strand to known CDS sequences in 12% of all sRNA candidates (Fig. 1A). The vast majority of all cDNAs represented tRNA and ribosomal RNA fragments (60%).

Cis‐regulatory elements in bacteria often harbour extended RNA secondary structures, which convey increased metabolic stability 30. The corresponding transcripts are of small size and hence are frequently detectable in small RNA libraries. Whether these are actual sRNAs or rather represent simple by‐product of the cellular turnover remains matter of debate 31. We included even these contigs in our survey for the identification of genomic regions, which would be suitable to design multiplex PCR assays. The resulting contigs were analysed *via* blast to identify candidates that are specific for MTB. Finally out of 3030 cDNA contigs, 80% of the isolated sRNA gene candidates are found to be specific to MTB genome (data not shown).

### IS6110, en masse with npcTB _6715, as the diagnostic markers for the mPCR assay

Multiplex PCRs to detect pathogens within patient samples demand species‐specific primer design for identification of the disease‐causing agent and also to discriminate against other yet closely related species 32, 33. Here, we evaluate sRNA genes as potential targets for PCR‐based amplification for diagnosis of MTB. The mPCR assay also compensates for drawbacks typically associated with bacterial cell culture and microscopy for the detection of MTB. The inclusion of additional targets provides increased selectivity to PCR‐based techniques.

Phylogenetic analysis *via* blast revealed that the npcTB_6715 candidate is detectable only within the mycobacterial complex (Supp. Dataset 1‐Alignment HitTable). The RNA overlaps partially the 5′ leader and CDS of hypothetical protein LH57_10405 (606 nts) (GenBank: AIR14655.1) (Fig. S1). The examination of RNA secondary structures *via* mfold 34 identified extended RNA stem loop structures. Given its genomic localization, the candidate might be leftover of *cis*‐regulatory circuits to control AIR14655.1 gene expression. However, its actual molecular functions are not known. The candidate was selected for PCR assays based on its sequence features, which allowed simple PCR design.

For long, IS*6110* has been target of choice because the multi‐copy sequence is specific for the mycobacterial complex 10. Recently however, various reports emphasized the emergence of IS*6110*‐negative MTB strains 15, 16. The addition of the sRNA gene as marker would aid in the detection of IS*6110*‐negative variants. So far, different regions of the IS*6110* were scrutinized for primer design and most often they relied on nucleotide positions 762–883 35. Here, we selected PCR primers from position 127–326 nts of the IS*6110*, which has not been explored for the design of diagnostic assays.

### Optimization of the mPCR assay

Multiplex PCR assays demand extensive optimization to achieve robust results. The annealing temperature, primer design, MgCl_2_ and dNTPs concentrations impact on the efficiency of PCR amplification. Analysis with gradient PCR revealed that for our assay primer annealing at 65.2°C is optimal and yields strongest amplification for all three targets (Fig. S2A–C).

#### Primer concentration

The parallel application of two primer pairs might cause unexpected interactions between both PCRs. We titrated primer concentrations in serial dilutions; all reactions were programmed with 1 ng of *M. tuberculosis* genomic DNA. Finally, 0.1 μM of IS*6110* and 0.5 μM of npcTB_6715 primers yielded balanced amplifications (Fig. S3A, red box).

#### The AIC internal control plasmid to identify PCR inhibitors

Depending on specific protocols for genomic DNA extraction sample contamination by PCR inhibitors must be assumed 36. We designed the ‘Amplification Internal Control’ (AIC) plasmid as internal control, which enables the detection of false negative results. Certainly, AIC concentrations have to be titrated extensively for identification of thresholds that ensure the amplification of control plasmids but minimize the inhibition of PCRs for other targets. A wide range of pL250 plasmid DNA concentrations (1000 ng to 0.1 fg per reaction) was subjected to PCR (Seegene, USA). The results revealed that concentrations of up to 10 pg AIC per reaction allow both the detection of effective control and target gene amplification (Fig. S3B, red box).

#### The titration of optimal dNTPs and MgCl_2_ concentrations

To determine the most effective dNTP concentration, serial dNTP dilutions ranging from 200 to 900 μM were analysed. The strongest amplification was observed with 0.4 mM dNTPs, as evident by gel electrophoresis (Fig. S4A). Similarly, we analysed the optimal MgCl_2_ concentration with serial dilutions ranging from 1.0 to 2.8 mM. Finally, 1.8 mM MgCl_2_ proved best and yielded strongest amplification of both IS*6110* and npcTB_6715 (Fig. S4B), respectively.

### Analytical specificity of the mPCR

**Figure 2:**
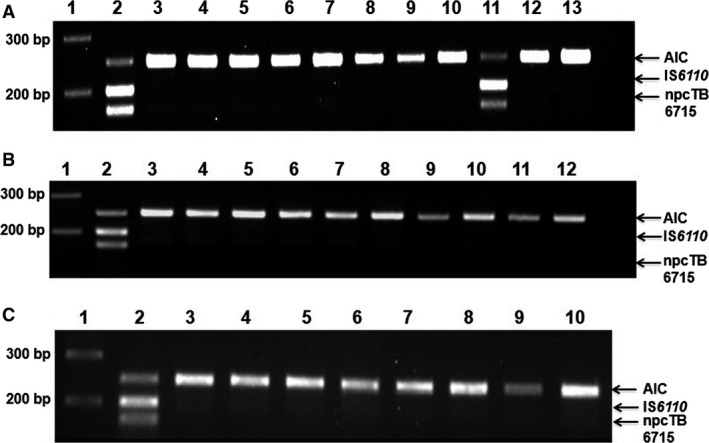
Specificity of the mPCR assay analysed with 4% agarose gel‐electrophoresis. The template DNA was 10 ng of MTB H37Rv genomic DNA per reaction. Primers for IS *6110* and npcTB_6715 were used at 0.1 μM and 0.5 μM, respectively. (**A**) MTB complex and NTM, Lane 1: 100 bp DNA ladder, Lane 2: MTB H37Rv, Lane 3: *M. avium*, Lane 4: *M. abscessus*, Lane 5: *M. fortuitum*, Lane 6: *M. fortuitum*, Lane 7: *M. gordonae*, Lane 8: *M. scroferaceum*, Lane 9: *M. intracellulare*. Lane 10: *M. kansasii*, Lane 11: MTB H37Ra, Lane 12: *M. malmoense*, Lane 13: Negative control (**B**) NTM and lower respiratory pathogens, Lane 1: 100 bp DNA ladder, Lane 2: MTB H37Rv, Lane 3: *M. marinum*, Lane 4: *M. chelonae*, Lane 5: *Staphylococcus aureus*, Lane 6: *Streptococcus pneumoniae*, Lane 7: *Klebsiella pneumoniae*, Lane 8: *Pseudomonas aeruginosa*, Lane 9: *Haemophilus influenza*. Lane 10: *Haemophilus parainfluenza*, Lane 11: *Moraxella catarrhalis*, Lane 12: Negative control (**C**) normal flora bacterial strains. Lane 1: 100 bp DNA ladder, Lane 2: MTB H37Rv, Lane 3: *Escherichia coli*, Lane 4: *Bifidobacterium bifidum*, Lane 5: *Corynebacteria sp*., Lane 6: *Lactobacillus sp*, Lane 7: *Neisseria meningitidis*, Lane 8: *Staphylococcus epidermidis*, Lane 9: *Coagulase Negative Streptococcus*, Lane 10: Negative control.

The specificity of the assay was validated with 10 closely related NTM strains as template. Individual strains were selected according to their prevalence within Asia to account for potential cross‐reaction with common targets 37. In addition, we included seven pathogens of the lower respiratory tract and seven microorganisms common to non‐diseased individuals. We only observed successful amplifications for MTB strains (MTB H37Rv and MTB H37Ra) (Fig. 2A–C). Under these test conditions, the results revealed 100% specificity. Thus, the assay specifically detects MTB under test conditions.

### Analytical sensitivity of the mPCR

**Figure 3:**
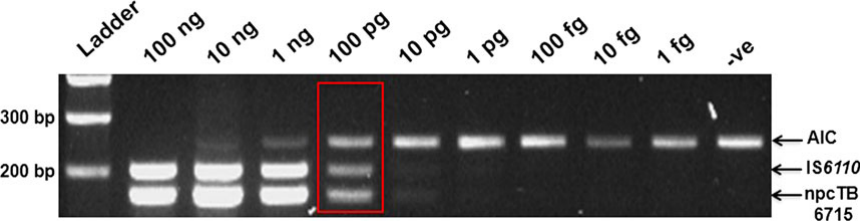
Sensitivity of the mPCR assay using different amounts of template genomic DNA of MTB H37Rv ranging from 100 ng to 1 pg per reaction analysed with 3% agarose gel‐electrophoresis. Primers for IS *6110* and npcTB_6715 were used at 0.1 μM and 0.5 μM, respectively. Ladder: 100 bp DNA ladder, ‐ve: Negative control.

The sensitivity of our mPCR assay was analysed with serial dilutions of MTB genomic DNA samples (100 ng to 1 pg). The actual detection threshold was about 100 pg genomic DNA per 20 μl total reaction, which is roughly equivalent to 10^3^ bacteria (one bacterium contains 10 fg genomic DNA) (Fig. 3, red box) 38. Apparently, with this sensitivity, the mPCR assay is about 10 times more sensitive than smear microscopy, which requires minimally 10,000 AFB/ml sample for the detection of MTB 39.

### Validation of the mPCR with culture positive samples

For this mPCR assay, samples that contain either the IS*6110* (200 nts) or npcTB_6715 (167 nts) amplicon deliver positive signals. To exclude false negatives, AIC (250 nts) supplement is mandatory. For validation and to test the mPCR with more diverse patient material, we utilized genomic DNA derived from 500 culture positive samples and compared our results with those of the ‘Mycobacterium CM line probe assay’ (LPA). The LPA test is based on reverse hybridization of PCR products to their complementary probes and allows the detection of different mycobacteria on (sub) species level.

With the above collection as template, the LPA analysis identified 371 MTB and 129 NTM samples. Among the latter, 124 samples represented various NTM isolates (41 *Mycobacterium abssessus*, 27 *Mycobacterium intracelulare*, 23 *Mycobacterium kansasii*, 11 *Mycobacterium fortuitum*, 7 *Mycobacterium avium*, 4 *Mycobacterium gordonae*, 4 *Mycobacterium scrofulaceum*, 3 *Mycobacterium chelonae*, 1 *Mycobacterium xenopi*, 1 *Mycobacterium ulcerance*, 1 *Mycobacterium marinum*, 1 *Mycobacterium malmoense* isolates); and finally 5 samples were classified as non‐mycobacterial species. These results were utilized as reference point to assess the validity of the mPCR design. Among the 500 samples analysed, 372 (74.4%) isolates were mPCR positive for MTB. A representative gel picture of the mPCR result was shown (Fig. S5). Interestingly, with the mPCR we did not uncover any IS‐negative isolate. Finally, the remaining 128 (25.6%) isolates were mPCR negative for MTB, as only the AIC displayed substantial PCR signals (which also indicates the absence of PCR inhibitors) (Table 1 [a]).

**Table 1:** Performance of mPCR (npcTB_6715 and IS*6110*) compared to that of the Line Probe assay (LPA) for the 500 culture positive samples

(a)	
Assay sensitivity	98.9%
Assay specificity	96.1%
Positive predictive value	98.6%
Negative predictive value	96.8%

(b)	mPCR positive	mPCR negative	Total
LPA positive	367	4	371
LPA negative	5	124	129
Total	372	128	500

Based on the LPA assay, five samples were found to be false positives and four samples were false negatives. Although in general the cause of false negative results might be the presence of PCR inhibitors 36, this is highly unlikely due to the successful amplification of the AIC for all 4 samples. Nevertheless, the cause of false negatives may be linked to the loss of MTB DNA during the extraction procedure. On the other hand, the false positives might be caused by contaminations of the amplicons or the stock culture of the NTM/MTB, or carry‐over during the PCR step.

Our mPCR assay reached sensitivity and specificity levels of 98.9% and 96.1%, respectively (Table 1 [b]). These values were comparable to those obtained by other assays based on PCR for the detection of MTB. Sensitivity and specificity of PCR assays vary widely, ranging from 63 to 100% and from 62 to 100%, respectively 40, 41, 42, 43, 44, 45. The differences in PCR sensitivities certainly depend on the PCR target 46. The high sensitivities of our assay substantiated the diagnostic potential of using the sRNA gene with IS*6110* as diagnostic biomarkers for MTB. The PPV and NPV for this mPCR assay are 98.6% and 96.8% respectively (Table 1 [b]). These results indicate that the mPCR assay is useful for the rapid identification of MTB in cultures.

## Conclusion

Collectively, our findings demonstrate that the multiplex PCR, amplifying npcTB_6715 sRNA gene and IS*6110*, has potential to detect MTB complex especially in culture positive isolates. The detection limit of the mPCR assay was 100 pg of genomic DNA per reaction. Our novel sRNA‐based mPCR assay may have clinical importance, whereby it can be use to differentiate between viable and non‐viable TB. The sensitivity and specificity of the mPCR assay were 98.4% and 96.1%, respectively, which underscore the potential of this assay for specific diagnosis of MTB. Moreover, the PPV and NPV values of 98.6% and 95.4% were comparable to that of the LPA. The mPCR assay is also cheaper and easier to handle because it involves only one amplification cycle compared to the 3 steps of LPA. Thus, the clinical potential of the npcTB_6715 was corroborated that it can be used alone or in concert with any target gene for the gene‐based detection of MTB.

## Author contribution

PK and LPL performed the experiments. TSR and THT conceived and designed the experiments. CAR, TSR and THT analysed the data. GR and ESC performed the clinical strain and supervised. PK, CM, SAA, CAR and THT wrote the study. PK, GR, CM, SAA, ESC, LPL, CAR, TSR and THT performed the final approval of the version to be submitted.

## Conflict of interest

The authors confirm that there are no conflict of interests.

## Supporting information


**Fig. S1** Depicted is the genomic blast organization of npcTB_6715 including flanking genes
**Fig. S2** Optimization of annealing temperature for IS*6110*, npcTB_6715 and pL250 primers. Gradient PCR were done for individual primer pair with the annealing temperature ranging from 62°C to 70°C, run on a 2% agarose gel. **(a) IS*6110* (b) npcTB_6715 (c) amplification internal control (AIC)**. Ladder: 100 bp DNA ladder.
**Fig. S3** (**A**) Optimization of primer concentrations for IS*6110* and npcTB_6715 targets in mPCR, run on a 3% agarose gel. The template DNA was 1 ng of *M. tuberculosis* H37Rv genomic DNA per reaction. In each lane, the primer concentrations were indicated respectively for IS6110 and npcTB_6715 (µM). Ladder: 100 bp DNA ladder, (**B**) Optimization of the amounts of AIC in the mPCR assay per reaction, run on a 3% agarose gel. Different amounts of AIC ranging from 1000 ng to 0.1 fg, 10 ng of *M. tuberculosis* H37Rv genomic DNA, 0.1 µM of IS*6110* primers, and 0.5 µM of npcTB_6715 primers were used in the reaction. Ladder: 100 bp DNA ladder
**Fig. S4** (**A**) Agarose gel electrophoresis of multiplex PCR using different concentrations of dNTPs ranging from 200 to 900 µM, run on a 3% agarose gel. The template DNA was 10 ng of MTB H37Rv genomic DNA per reaction. Primers for IS*6110* and npcTB_6715 were used at 0.1 µM and 0.5 µM, respectively. Ladder: 100 bp DNA ladder. (**B**) Optimization of the amount of MgCl_2_ ranging from 1.0 to 2.8 mM, run on a 3% agarose gel. The template DNA used was 10 ng of MTB H37Rv genomic DNA per reaction, with 0.1 µM of IS*6110* primers and 0.5 µM of npcTB_6715 primers. Ladder: 100 bp DNA ladder
**Fig. S5** Representative gel picture of multiplex PCR products derived from culture positive samples, analyzed with 4% agarose gel‐electrophoresis. M: 100 bp DNA ladder. Lane 1: Negative control, Lane 2: Positive control, Lane 3‐17: 15 culture positive samples.
**Table S1** Primers used in the mPCR
**Table S2** Bacterial strains for specificity testing
