# Supplementary material for: RNomic identification and evaluation of npcTB_6715, a non‐protein‐coding RNA gene as a potential biomarker for the detection of Mycobacterium tuberculosis
Source: J Cell Mol Med. 2017 Jul 29;21(10):2276–83. doi: 10.1111/jcmm.13148 (PMC5618688; doi:10.1111/jcmm.13148)
Supplement: Supplementary file 1 — Fig. S1 Depicted is the genomic blast organization of npcTB_6715 including flanking genes Fig. S2 Optimization of annealing temperature for IS6110, npcTB_6715 and pL250 primers. Gradient PCR were done for individual primer pair with the annealing temperature ranging from 62°C to 70°C, run on a 2% agarose gel. (a) IS6110 (b) npcTB_6715 (c) amplification internal control (AIC). Ladder: 100 bp DNA ladder. Fig. S3 (A) Optimization of primer concentrations for IS6110 and npcTB_6715 targets in mPCR, run on a 3% agarose gel. The template DNA was 1 ng of M. tuberculosis H37Rv genomic DNA per reaction. In each lane, the primer concentrations were indicated respectively for IS6110 and npcTB_6715 (µM). Ladder: 100 bp DNA ladder, (B) Optimization of the amounts of AIC in the mPCR assay per reaction, run on a 3% agarose gel. Different amounts of AIC ranging from 1000 ng to 0.1 fg, 10 ng of M. tuberculosis H37Rv genomic DNA, 0.1 µM of IS6110 primers, and 0.5 µM of npcTB_6715 primers were used in the reaction. Ladder: 100 bp DNA ladder Fig. S4 (A) Agarose gel electrophoresis of multiplex PCR using different concentrations of dNTPs ranging from 200 to 900 µM, run on a 3% agarose gel. The template DNA was 10 ng of MTB H37Rv genomic DNA per reaction. Primers for IS6110 and npcTB_6715 were used at 0.1 µM and 0.5 µM, respectively. Ladder: 100 bp DNA ladder. (B) Optimization of the amount of MgCl2 ranging from 1.0 to 2.8 mM, run on a 3% agarose gel. The template DNA used was 10 ng of MTB H37Rv genomic DNA per reaction, with 0.1 µM of IS6110 primers and 0.5 µM of npcTB_6715 primers. Ladder: 100 bp DNA ladder Fig. S5 Representative gel picture of multiplex PCR products derived from culture positive samples, analyzed with 4% agarose gel‐electrophoresis. M: 100 bp DNA ladder. Lane 1: Negative control, Lane 2: Positive control, Lane 3‐17: 15 culture positive samples. Table S1 Primers used in the mPCR Table S2 Bacterial strains for specificity testing [file JCMM-21-2276-s001.doc]

**Supplemental Material**

**
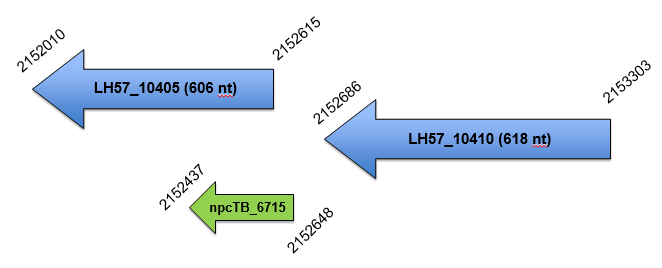
**

**Fig. S1 Depicted is the genomic blast organization of npcTB_6715 including flanking genes.**

**
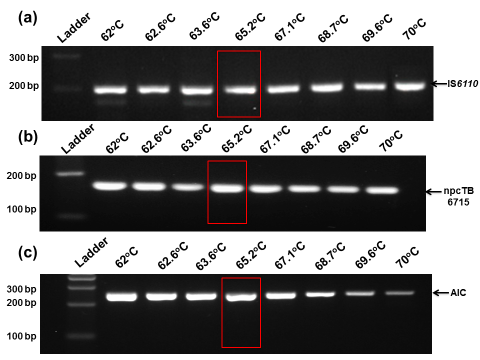
**

**Fig. S2 Optimization of annealing temperature for IS*6110*, npcTB_6715 and pL250 primers.** Gradient PCR were done for individual primer pair with the annealing temperature ranging from 62°C to 70°C, run on a 2% agarose gel. **(A) IS*6110* (b) npcTB_6715 (C) amplification internal control (AIC).** Ladder: 100 bp DNA ladder.


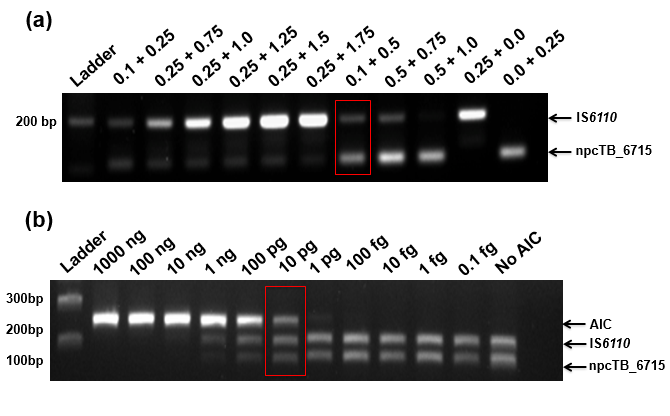


**Fig. S3 (A) Optimization of primer concentrations for IS*6110* and npcTB_6715 targets in mPCR, run on a 3% agarose gel.** The template DNA was 1 ng of *M. tuberculosis* H37Rv genomic DNA per reaction. In each lane, the primer concentrations were indicated respectively for IS6110 and npcTB_6715 (µM). Ladder: 100 bp DNA ladder, **(B)** **Optimization of the amounts of AIC in the mPCR assay per reaction, run on a 3% agarose gel.** Different amounts of AIC ranging from 1000 ng to 0.1 fg, 10 ng of *M. tuberculosis* H37Rv genomic DNA, 0.1 µM of IS*6110* primers, and 0.5 µM of npcTB_6715 primers were used in the reaction. Ladder: 100 bp DNA ladder.


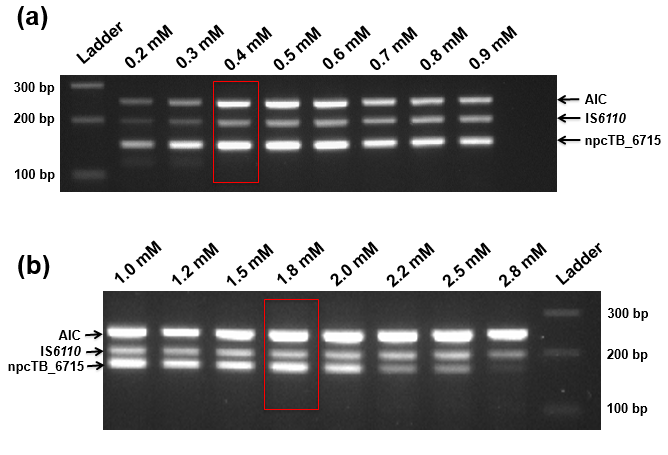


**Fig. S4 (A) Agarose gel electrophoresis of multiplex PCR using different concentrations of dNTPs ranging from 200 to 900 µM, run on a 3% agarose gel.** The template DNA was 10 ng of MTB H37Rv genomic DNA per reaction. Primers for IS*6110* and npcTB_6715 were used at 0.1 µM and 0.5 µM, respectively. Ladder: 100 bp DNA ladder. **(B)** **Optimization of the amount of MgCl_2_ ranging from 1.0 to 2.8 mM, run on a 3% agarose gel.** The template DNA used was 10 ng of MTB H37Rv genomic DNA per reaction, with 0.1 µM of IS*6110* primers and 0.5 µM of npcTB_6715 primers. Ladder: 100 bp DNA ladder.


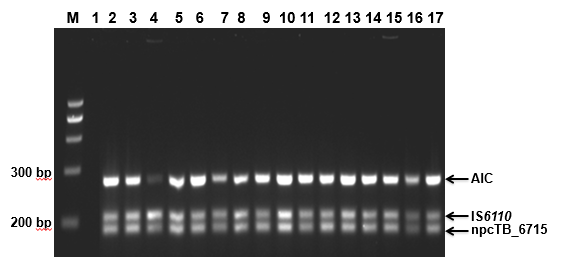


**Fig. S5 Representative gel picture of multiplex PCR products derived from culture positive samples, analyzed with 4% agarose gel-electrophoresis.** M: 100 bp DNA ladder. Lane 1: Negative control, Lane 2: Positive control, Lane 3-17: 15 culture positive samples.

**Table S1.** Primers used in the mPCR

| **Target** | **Primer name**  **Sequence (5’-3’)** | **Amplicon size (bp)** |
| --- | --- | --- |
| npcTB_6715 sRNA gene | npc-F ATCGACAGTGGCGCACGCATT  npc-R CATCGCCGGTAGGCGTTTCG | 167 |
| IS*6110* | IS-F CGCGGTCAGCACGATTCGGA  IS-R TCGCGTTCGCCCTTCGCAAT | 200 |
| pL250 (AIC) | pL-F GTCTACCAGGCATTCGCTTCAT  pL-R CTGTGAATGCTGCGACTACGAT | 250 |

**Table S2. Bacterial strains for specificity testing**

| **Bacterial strains** | **Description** |
| --- | --- |
| ***Mycobacterium tuberculosis* H37Rv** | Virulent laboratory strain |
| ***Mycobacterium tuberculosis* H37Ra** | Attenuated tubercle bacillus closely related *M. tuberculosis* H37Rv |
| ***Mycobacterium avium*** | Non tuberculosis Mycobacterium |
| ***Mycobacterium abscessus*** | Non tuberculosis Mycobacterium |
| ***Mycobacterium fortuitum*** | Non tuberculosis Mycobacterium |
| ***Mycobacterium gordonae*** | Non tuberculosis Mycobacterium |
| ***Mycobacterium scroferaceum*** | Non tuberculosis Mycobacterium |
| ***Mycobacterium intracellulare*** | Non tuberculosis Mycobacterium |
| ***Mycobacterium kasasii*** | Non tuberculosis Mycobacterium |
| ***Mycobacterium malmoense*** | Non tuberculosis Mycobacterium |
| ***Mycobacterium marinum*** | Non tuberculosis Mycobacterium |
| ***Mycobacterium chelonae*** | Non tuberculosis Mycobacterium |
| ***Staphylococcus aureus*** | Lower respiratory tract pathogen |
| ***Streptococcus pneumoniae*** | Lower respiratory tract pathogen |
| ***Klebsiella pneumoniae*** | Lower respiratory tract pathogen |
| ***Pseudomonas aeruginosa*** | Lower respiratory tract pathogen |
| ***Haemophilus influenza*** | Lower respiratory tract pathogen |
| ***Haemophilus parainfluenza*** | Lower respiratory tract pathogen |
| ***Moraxella catarrhalis*** | Lower respiratory tract pathogen |
| ***Escherichia coli*** | Normal flora |
| ***Bifidobacterium bifidum*** | Normal flora |
| ***Corynebacterium* sp.** | Normal flora |
| ***Lactobacillus sp.*** | Normal flora |
| ***Neisseria meningitidis*** | Normal flora |
| ***Staphylococcus epidermidis*** | Normal flora |
| **Coagulase Negative *Streptococcus*** | Normal flora |
